# Apparent Diffusion Coefficient Can Predict Therapy Response of Hepatocellular Carcinoma to Transcatheter Arterial Chemoembolization

**DOI:** 10.1159/000520716

**Published:** 2021-11-08

**Authors:** Ralph Drewes, Constanze Heinze, Maciej Pech, Maciej Powerski, Katja Woidacki, Andreas Wienke, Alexey Surov, Jazan Omari

**Affiliations:** ^a^Department of Radiology and Nuclear Medicine, Otto-von-Guericke University, Magdeburg, Germany; ^b^2nd Department of Radiology, Medical University of Gdansk, Gdansk, Poland; ^c^Section Experimental Radiology, Department of Radiology and Nuclear Medicine, Otto-von-Guericke University, Magdeburg, Germany; ^d^Institute for Medical Epidemiology, Biometrics and Informatics, Martin-Luther-University Halle Wittenberg, Halle, Germany

**Keywords:** Hepatocellular carcinoma, Transcatheter arterial chemoembolization, Magnetic resonance imaging, Apparent diffusion coefficient, Treatment response

## Abstract

**Aim:**

The goal of this meta-analysis was to assess the apparent diffusion coefficient (ADC) as a pre- and posttreatment (ADC value changes [ΔADC]) predictive imaging biomarker of response to transcatheter arterial chemoembolization (TACE) in patients with hepatocellular carcinoma (HCC).

**Methods:**

Scopus database, Embase database, and MEDLINE library were scanned for connections between pre- and posttreatment ADC values of HCC and response to TACE. Six studies qualified for inclusion. The following parameters were collected: authors, publication year, study design, number of patients, drugs for TACE, mean ADC value, standard deviation, measure method, *b* values, and Tesla strength. The Quality Assessment of Diagnostic Studies 2 instrument was employed to check the methodological quality of each study. The meta-analysis was performed by utilizing RevMan 5.3 software. DerSimonian and Laird random-effects models with inverse-variance were used to regard heterogeneity. The mean ADC values and 95% confidence intervals were computed.

**Results:**

Six studies (*n* = 271 patients with 293 HCC nodules) were included. The pretreatment mean ADC in the responder group was 1.20 × 10<sup>−3</sup> mm<sup>2</sup>/s (0.98, 1.42) and 1.14 × 10<sup>−3</sup> mm<sup>2</sup>/s (0.89, 1.39) in the nonresponder group. The analysis of post-TACE ΔADC revealed a threshold of ≥20% to identify treatment responders. No suitable pretreatment ADC threshold to predict therapy response or discriminate between responders and nonresponders before therapy could be discovered.

**Conclusion:**

ΔADC can facilitate early objective response evaluation through post-therapeutic ADC alterations ≥20%. Pretreatment ADC cannot predict response to TACE.

## Introduction

Most hepatocellular carcinoma (HCC) patients are ineligible for curative resection at the time of diagnosis due to various reasons: multifocal HCC, vascular infiltration, impaired liver function, and extrahepatic tumor manifestations [1, 2, 3, 4, 5, 6]. Transcatheter arterial chemoembolization (TACE) became an established, repeatable, stage-dependent therapy for patients unsuitable for surgery or patients awaiting transplantation (i.e., bridging). This therapy incorporates the emulsification of chemotherapeutic agents in a drug carrier and embolic material, which are injected into the tumor feeding arteries, leading to tumor necrosis and ultimately tumor regression [7]. For conventional TACE (cTACE), chemotherapeutic agents such as doxorubicin or cisplatin are blended with embolic agents (e.g., Gelfoam or polyvinyl alcohol particles) and Lipiodol that represents the drug carrier. Drug-eluting beads (DEBs) have been imposed as a novel drug carrier (polyvinyl alcohol microspheres), allowing higher concentration of the drug within the target lesion and at the same time, lower systemic concentration, facilitating similar outcomes with less toxicity than cTACE [8, 9, 10, 11, 12]. DEB-TACE and cTACE both lead to ischemic necrosis of the target via cytotoxic and ischemic mechanisms.

Evaluation of tumor response remains paramount since lack of response would necessitate an alternative treatment approach (e.g., systemic treatment). HCCs demonstrating partial response to TACE might show a further response after repeated TACE [13]. The earliest time however to appraise HCC response to both DEB-TACE and cTACE is 30–90 days after treatment as early contrast enhancement, e.g., gadolinium-enhanced magnetic resonance imaging (MRI), enables no differentiation between residual tumor and post-therapeutic inflammatory effects [14, 15, 16]. Therefore, diffusion-weighted imaging (DWI) came into play, which facilitates the detection of residual neoplastic tissue as well as necrotic areas in a timely fashion [17]. Apparent diffusion coefficient (ADC) enables quantitative assessment and the velocity of Brownian molecule movement within the interstitial space [14, 15], and in experimental studies, a strong inverse correlation between ADC and cell count/degree of cellularity was found [18, 19]. However, clinical data for significant correlations between ADC and cellularity in various tumors are inconsistent; more precisely, in meta-analysis of large patient data, different inverse correlations were identified according to the tumor entity, e.g., strong correlation in gliomas, ovarian cancer, and lung cancer versus low correlation for lymphomas [20].

Several studies have evaluated DWI for HCC patients after TACE treatment [13, 17, 21, 22, 23]. Few investigators demonstrated that the mean tumor ADC values tend to increase after TACE corresponding to tumor necrosis [13, 21, 24].

The desire to find an earlier response assessment tool rather than a mere evaluation of size and enhancement (RECIST and mRECIST) led to further examinations of the ADC. The present meta-analysis scrutinizes ADC as a predictor of tumor response to TACE in HCC patients.

## Methods

An IRB approval was not required for this meta-analysis.

### Data Acquisition

**Fig. 1:**
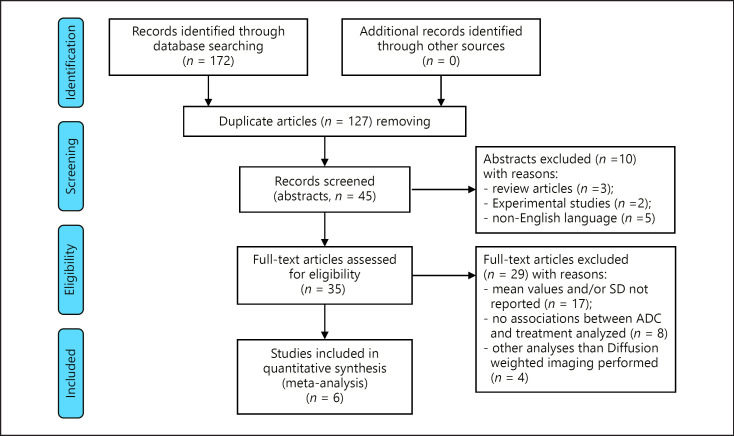
PRISMA flowchart of the data acquisition. ADC, apparent diffusion coefficient; DWI, diffusion-weighted imaging.

MEDLINE library, Embase database, and Scopus database were scanned for connections between pretreatment ADC values of HCC and treatment response to TACE up to September 2021 (Fig. 1). The PRISMA statement was employed for research [25].

The following terms were searched: “DWI OR diffusion weighted imaging OR ADC OR apparent diffusion coefficient AND HCC OR hepatocellular carcinoma AND TACE OR transcatheter arterial chemoembolization.” Secondary references were also recruited. The primary search revealed 172 results. Duplicate articles (*n* = 127) were excluded. Abstracts of the remaining 45 articles were scrutinized. Furthermore, review articles, in vitro and experimental animal studies, case reports, and non-English publications were excluded (*n* = 10). Other articles (*n* = 29) were excluded for different reasons: mean ADC values or standard deviation not reported, no associations between ADC and treatment analyzed, or other analyses than DWI were performed.

Finally, 6 studies were eligible for the present meta-analysis [26, 27, 28, 29, 30, 31]. The following data were used from the literature: authors, year of publication, number of patients, and reported ADC values of HCC in responders and nonresponders.

### Meta-Analysis

**Fig. 2:**
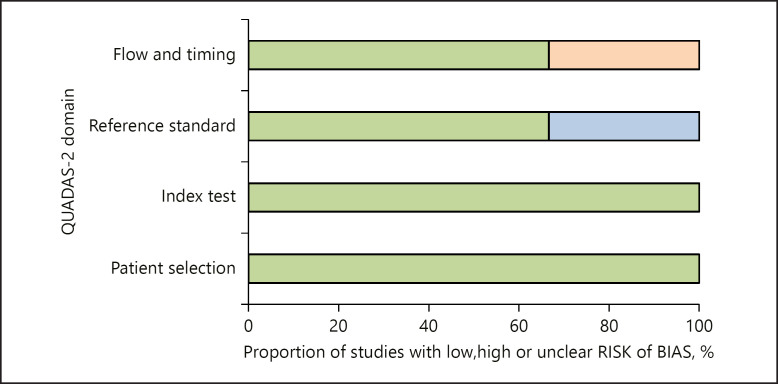
QUADAS-2 quality assessment of the included studies. QUADAS-2, Quality Assessment of Diagnostic Studies 2.

In the first step, the methodological quality of the 6 included studies were checked by one observer (A.S.), applying the Quality Assessment of Diagnostic Studies instrument [32] (Fig. 2).

Afterward, the meta-analysis was done by using RevMan 5.3 (computer software, version 5.3; the Nordic Cochrane Centre, Copenhagen, Denmark, the Cochran Collaboration, 2014). Heterogeneity was computed by means of the inconsistency index *I*^2^ [33, 34]. Last, DerSimonian and Laird [35] random-effects models with inverse-variance weights were employed without any further correction. The primary objectives were to evaluate the use of pretreatment ADC to predict response to TACE and ADC value changes (ΔADC) as an indicator of response to TACE.

## Results

Six studies were included in this meta-analysis [26, 27, 28, 29, 30, 31]. The studies come from the USA, China, and Japan and were reported between 2010 and 2020 (Table 1), where 4 studies are prospective and 2 retrospective.

All studies included patients with unresectable HCC treated with cTACE (*n* = 4) or DEB-TACE (*n* = 2). Response assessment using dynamic contrast enhancement MRI was evaluated by WHO, RECIST 1.1, EASL, and mRECIST criteria as well as DWI. A descriptive statistical analysis including tumor distribution, ECOG performance status, Child-Pugh class, and Barcelona Clinic Liver Cancer staging was performed by Kokabi et al. [26], Ou et al. [30], and Yuan et al. [31], but the other studies lacked or did not publish this data.

Noteworthy information for the analyzed studies is listed below:

Kokabi et al. [26] concluded ΔADC ≥20% has 100% sensitivity and specificity in predicting therapy response according to mRECIST and EASL at 1 and 3 months. Prediction of HCC relapse was analyzed for a ΔADC threshold of 13.6% (sensitivity 76.9% and specificity 100%) of Kubota et al. [27].

Lin et al. [28] as well as Yuan et al. [31] included only histopathologically confirmed HCC. A maximum tumor diameter >2 cm and no large necrosis were inclusion criteria for Lin et al. [28].

In the study of Mannelli et al. [29], therapy-induced necrosis, depictable in ADC post-therapy, was correlated with histopathological examinations of all transplanted patients (15 of 36 included patients who underwent liver transplantation at some point after TACE). An overall amount of 293 HCC lesions were treated with TACE: 182 lesions were categorized as responders and 111 as nonresponders. Only half of the studies declared the mean lesion sizes. Viral hepatitis was the underlying etiology for HCC in 160/271 patients; however, data regarding etiology is not published for 85 patients (see Table 1).

Different MRI scanners and *b* values were used. Four studies employed a 1.5 Tesla MRI, one study a 3 Tesla MRI, and only one study by Lin et al. [28] neither gave information about the MRI manufacturer nor about Tesla strength (Table 1). DWI *b* values were reported by all studies. The *b* values were as follows: b50, 400, 800 (Kokabi et al. [26]); b0, 500 (Kubota et al. [27]); b0, 20, 50, 100, 200, 400, 600, 800, 1,000 (Lin et al. [28]); b0, 50, 500 (Mannelli et al. [29]); b0, 400 (Ou et al. [30]); and b0, 800, 1,500, 2,000 (Yuan et al. [31]).

DWI was acquired by a breath-holding technique. ADC values were measured with manual whole-lesion region of interest placement.

The timeframe of MRI acquisition pre- and post-TACE was heterogenous across included studies: Kokabi et al. [26] (pre: no data, post: 3 h, 1 and 3 months), Kubota et al. [27] (pre: 1–2 days, post: 5–7 days), Lin et al. [28] (pre: 1–3 days, post: 1 month), Mannelli et al. [29] (within 3 months before and after TACE), Ou et al. [30] (pre: 0–2 days, post: 1, 2 and 4 weeks), and Yuan et al. [31] (pre: no data, post: 1 month, every 3 months). The TACE drugs, which were administered to the patients, were also different (Table 1).

**Fig. 3:**
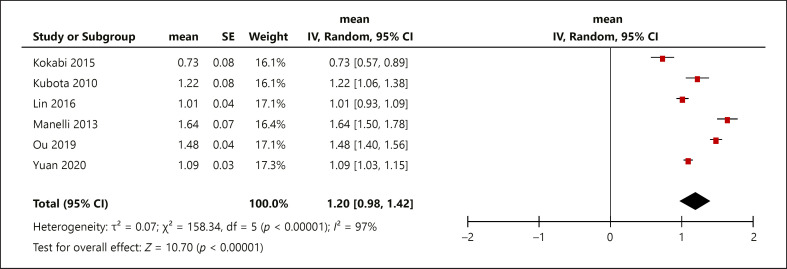
Responder mean ADC, standard deviation, heterogeneity, confidence interval, and whisker plots. ADC, apparent diffusion coefficient.

**Fig. 4:**
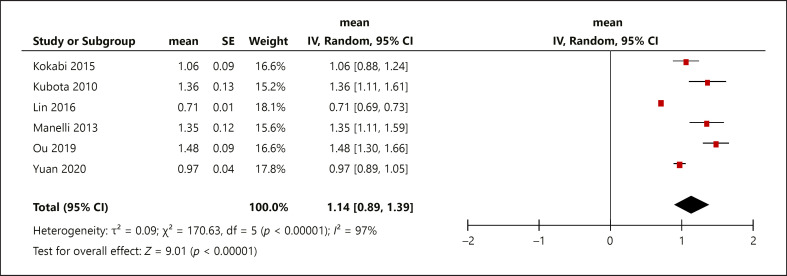
Nonresponder mean ADC, standard deviation, heterogeneity, confidence interval, and whisker plots. ADC, apparent diffusion coefficient.

**Fig. 5:**
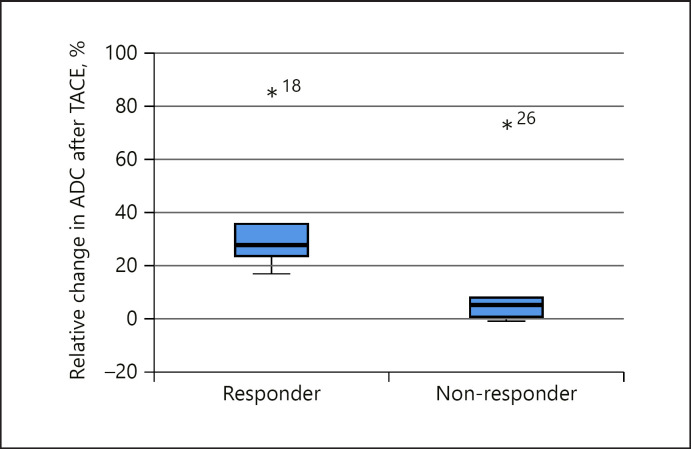
ADC box plots of responders and nonresponders to TACE. ADC, apparent diffusion coefficient; TACE, transcatheter arterial chemoembolization.

The pretreatment mean ADC for HCC lesions responding to TACE was 1.20 × 10^−3^ mm^2^/s (0.98, 1.42) and for the HCC lesions not responding to TACE 1.14 × 10^−3^ mm^2^/s (0.89, 1.39) (Fig. 3, 4). The box plots demonstrate the overlap between ADC values of responders and nonresponders (Fig. 5), indicating that pretreatment ADC values cannot predict treatment response to TACE.

**Fig. 6:**
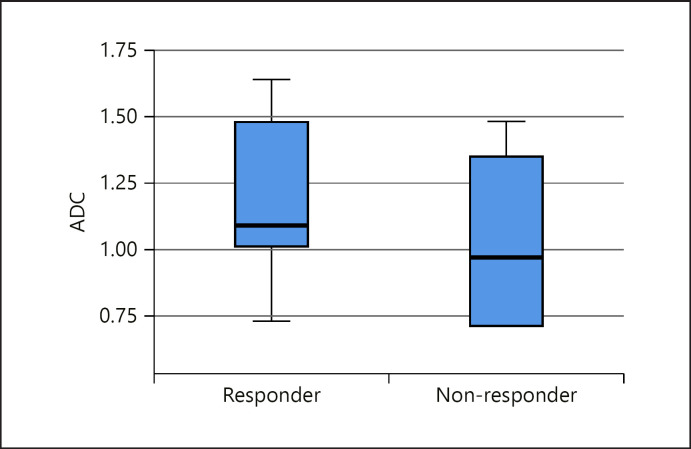
ΔADC box plots of responders and nonresponders to TACE. ΔADC, apparent diffusion coefficient value change; TACE, transcatheter arterial chemoembolization.

A second analysis of posttreatment ΔADC revealed a practical threshold of ≥20% to identify TACE responders (Fig. 6) with a statistical outlier resulting from the ADC values of Lin et al. [28] (Table 2). The same 6 studies were eligible for this second analysis.

## Discussion

This meta-analysis examines ADC values as a predictor of pathological response to transcatheter chemoembolization in HCC patients. TACE became the first choice of nonsurgical treatment [36, 37] for HCC patients with an intermediate Barcelona Clinic Liver Cancer stage with an estimated survival of 20 months [38]. TACE can cause some degree of ischemic liver damage as well as ischemic tumor necrosis, potentially leading to hepatic decompensation in a setting of coexisting cirrhosis [39, 40]. DEB-TACE has significantly lowered liver toxicity and systemic drug exposure than cTACE [41, 42].

Early objective tumor response evaluation remains a crucial aspect of any oncological treatment regime. Several authors have shown WHO and RECIST criteria to be unsuitable for HCC therapy response assessment [26]. Decrease in tumor size, measured by RECIST, lags behind changes in tumor enhancement, and ADC can take about 6 months to be measurable in response assessment.

In contrast, enhancement or necrosis-based response criteria (EASL and mRECIST) have displayed significant correlations with overall survival (OS) and progression-free survival in HCC after TACE [15, 43] and are independent predictors of survival after 1 and 3 months objective response [15, 44]. Recurrence after TACE occurs after therapy-induced ischemia and a resulting upregulation of angiogenesis and VEGF expression peaking 24–36 h after embolization [42, 45]. A decrease in contrast enhancement indicates a reduction or even interruption of tumor blood supply and correlates with necrosis and improved OS in HCC patients [23, 46, 47]. However, early enhancement patterns have clear limitations and cannot differentiate objectively between residual tumor, fibrous tumor capsule, inflammatory response (reactive edema), granulation tissue, or coagulative hemorrhagic necrosis [15, 16], sometimes even resulting in transiently increasing tumor diameters. High T1 pre-contrast intensity further complicates imaging interpretation.

Therefore, conclusive assessment of tumor response should be paramount before subjecting the patient to repeated TACE or alternative percutaneous procedures. A more immediate and objective assessment after TACE to evaluate response, predict further treatment success, and generate or adapt a robust and cost- and side-effect-efficient therapy plan appears tempting.

The predictive power of pretreatment ADC was examined previously for colorectal cancer and gastric carcinoma liver metastases [51] [48] as well as for esophageal cancer, head and neck carcinoma, and breast cancer [49, 50, 51, 52]. DWI sheds light on cellularity, tumor viability, tissue perfusion, and necrosis at any given time; an intact tumor cell membrane restricts water molecule diffusion (decreased ADC value) and increased permeability or lysis of the tumor cell membrane caused by tumor necrosis alleviates water molecule diffusion (higher ADC value) [53]. Hepatic cirrhosis has been demonstrated to also restrict diffusion secondary to reactive liver tissue fibrosis, leading to lowered mean ADC values for “healthy” or cancer-free hepatic tissue [54, 55]. Another ADC influencing factor could be the dual hepatic blood supply, especially in cases of infiltrative HCC (7–15% of all cases) with portal-vein thrombosis and a median OS of 5 months [56].

However, several studies have investigated the role of ADC in HCC; for instance, Jing et al. [57] found ADC value combined with tumor size can be used as a noninvasive method for preoperative evaluation of HCC. Besides, ADC has also been evaluated as a predictor of response to therapy, such as cisplatin-based hepatic arterial infusion chemotherapy [58] or stereotactic ablative radiotherapy [59].

The prognostic role of ADC was also investigated for various other tumors however with differing results even for the same entity: for instance, Ho et al. [60] found ADC to be a useful predictor of the outcome in cervical cancer following chemoradiation, whereas Meyer et al. [61] showed in their meta-analysis that ADC values alone were not reliable to predict therapy response to radiochemotherapy. These findings stress the need for further prospective studies taking both technical and clinical factors into consideration.

Several studies examine the application of DWI in patients treated with DEB-TACE [41, 62], cTACE, and radioembolization [13, 23, 24, 29, 50, 51, 62, 63, 64]. However, no consensus exists concerning ideal *b* values, time intervals between TACE and follow-up, or MRI field strength. Kokabi et al. [26] reported significantly elevated ADC values as early as 3 h post-DEB-TACE in HCC patients and suggested a threshold of 20% immediately post-TACE to predict responders with a 100% sensitivity and specificity. Some studies indicated that a higher percent increase in ADC levels after TACE predicts responder lesions and lack of recurrence [64, 65, 66], whereas ADC values of nonresponder lesions barely rise, if at all. Conflicting reports are published concerning the predictive power of pretreatment ADC values with a slight tendency toward the assumption that HCCs with lower pretreatment ADC values more likely respond to TACE [26, 67]. Correspondingly, published ADC value thresholds predicting objective response and nonresponse vary significantly across studies. Kokabi et al. [67] reported an ADC value threshold of 0.83 × 10^−3^ mm^2^/s, below which an objective response can be predicted (91% sensitivity and 96% specificity) at 1 and 3 months after DEB chemoembolization. In contrast, different threshold were reported by Mannelli et al. [29], 0.695 × 10^−3^ mm^2^/s; Yuan et al. [31], 1.618 × 10^−3^ mm^2^/s; and Dong et al. [64], 1.3 × 10^−3^ mm^2^/s.

Lower ADC values, and thus, higher cellularity, indicate an increased cell division rate and a higher susceptibility to antineoplastic agents such as doxorubicin [67]. Viable tumor cells in rather necrotic tumor areas remain difficult to eradicate due to poor perfusion and acidic and hypoxic microenvironments, leading to limited efficacy of doxorubicin or any other antineoplastic drug [68]. Lower levels of cellularity, i.e., higher ADC values, have also been associated with diminished response to chemotherapy and ischemia-inducing therapies [62]. Unfortunately, no clear correlation between ADC values and histological tumor grading for HCC has been established yet [67]; one study showed that ADC changes are significantly associated with histopathological tumor necrosis [63].

Highly vascular HCC lesions, promising targets for intra-arterial therapy, are found to have lower ADC values (more restricted diffusion), whereas higher ADC levels indicate pretreatment areas of necrosis, loss of cell membrane integrity, and tumor aggressiveness [29, 31, 64]. A strong correlation was found between post-TACE ADC and necrosis based on pathology and imaging [29]. Increased ADC values post-therapy were demonstrated to be associated with cellular edema, necrosis, apoptosis, and fibrosis [69].

The analysis of HCC patients treated with TACE failed to reveal a practical pretreatment ADC threshold to predict treatment responders (Fig. 5). Responder and nonresponder ADC values show a significant overlap; hence, no general conclusion can be drawn concerning the obviously heterogenous pretreatment ADC values. Possible explanations for failure to predict response (pretreatment) are dissimilar TACE drugs and varying ADC acquisition parameters.

However, ADC values post-TACE tell a different story. An increase of at least 20% in ADC values post-TACE clearly differentiates responders and nonresponders (Fig. 6) with a statistical outlier originating from one study (Lin et al. [28], Table 2). Lin et al. [28] published neither the MRI manufacturer nor field strength and used a variety of different TACE drugs (Table 1). Ultimately, the reason for the obvious discrepancy of these results compared to the other studies remains unknown and speculative. Apart from this outlier based on one study, the ΔADC metric remains robust despite different timeframes of ADC acquisition, different drugs, and scanners. As mentioned above, Kokabi et al. [26] observed an increase of ADC values in responders as early as 3 h post-TACE. Correspondingly, Bonekamp et al. [69] demonstrated response to TACE evaluation by increased ADC values in HCC patients.

The *b* values were different in the studies comprised in this meta-analysis, which results inherently in different ADC mean values as well. Furthermore, ADC values acquired on unequal MRI scanners and variable field strength (1.5 and 3 Tesla) are not simply interchangeable. These factors illustrate the main concerns of some controversies concerning the use of ADC values as biomarkers. ADC remains a composite coefficient affected by both perfusion and diffusion. The perfusion or flow sensitivity of ADC can be reduced with choosing more than one *b* value and a higher maximum value [63].

DWI technique, interobserver reproducibility, and reliability have been proven in several studies [70, 71]. DWI requires no administration of intravenous contrast agent and facilitates safe and comfortable examinations even for patients suffering from renal insufficiency. Clear correlations between histology and ADC values are still lacking in HCC patients but would provide a more objective insight into any factors contributing to different ADC values which are otherwise based on assumptions.

The findings of this study appear promising enough to warrant further examinations of ADC in a larger prospective study. ADC change offers a reliable and very early assessment of therapy success after TACE and facilitates efficient treatment alterations for nonresponders. Pretreatment ADC values and a predictive potential in HCC patients should be reevaluated with homogenous acquisition parameters to support or finally reject ideas of pretreatment predictions.

The present study has several limitations. Only a small number of studies met the inclusion criteria for pretreatment ADC analysis (6 studies) and delta ADC analysis (4 studies). Results in the literature form the basis of this analysis, bearing the possibility of a publication bias attributable to the general tendency to prefer and publish significant and positive results and to reject insignificant or negative ones. Some studies were excluded due to important missing metrics, e.g., standard deviation or clear group separation (responder and nonresponder).

The lack of standardized ADC acquirement and interpretation presents another major obstacle not only for HCC but also for any tumor entity. Many different variables, mostly technical MRI specifics, such as MR scanner, sequencing, *b* values, timeframes, and biological factors, contribute to ADC values and thresholds to an unknown degree [67]. Ultimately, a uniform and clinically applicable ADC threshold might only be adopted if most or all contributing factors are standardized so that DWI can unfold its true potential as an imaging (predictive) biomarker. Obvious difficulties specific to TACE and other intra-arterial interventions concerning comparability arise due to the subjective tumor targeting through tumor blushing, sluggish flow, stasis, and variable application of the drug type and dosage.

In conclusion, a threshold of elevated ADC values ≥20% post-TACE might be recommended to identify responders in HCC patients; the predictive power of pretreatment ADC values remains uncertain due to different findings across studies and must be reevaluated in a larger prospective trial with homogenous acquisition parameters.

## Statement of Ethics

An ethics statement is not applicable because this study is based exclusively on published literature.

## Conflict of Interest Statement

The authors have no conflict of interest to declare.

## Funding Sources

No funding was received for this article.

## Author Contributions

A.S. and A.W. conceived and designed the study; A.S., M.P., M.P., J.O., K.W., and C.H. contributed to collection and analysis of data; all the authors contributed to manuscript writing; C.H., M.P., and A.W. contributed to critical revision of the manuscript. All the authors approved the final manuscript submitted.

## Data Availability Statement

All data generated or analyzed during this study are included in this article. All research data are publicly available. Further inquiries can be directed to the corresponding author.

## Figures and Tables

**Table 1:** Overview of included Studies

Author	Included number of patients and etiology of hepatocellular cancer, HBV, and HCV	Included lesions, *n*	Responder	Non-responder	MR scanner	*b* values	ADC measure	Time for response/follow-up	TACE drug
Kokabi et al. [26]	Total, 12; HCV, 7/12; alcohol 3/12; HBV, 1/12; and other 1/12	12	6	6	Siemens 1.5 T	50, 400, 800	Manual ROI	3 h, 1 and 3 months	Doxorubicin beads (DEB) up to 100 mg

Kubota et al. [27]	Total, 25; no data regarding etiology, presumably HBV	36	23	13	GE medical 1.5 T	0, 500	Manual ROI	5–7 days MRI, 3 months CT	1–5 mL Lipiodol, 10–30 mg epirubicin hydrochloride

Lin et al. [28]	Total, 118; HBV, 66/118; no further data	118	67	51	No data	0, 20, 50, 100, 200, 400, 600, 800, 1,000	Manual ROI	1 month	Emulsion: 6 mg mitomycin: 20 mg Adriamycin: 60 mg cisplatin: 5–25 mL Lipiodol

Mannelli et al. [29]	Total, 36; HCV, 26/36; HBV, 47 7/36; HCV and HBV, 2/36; and alcohol, 1/36		34	13	Siemens 1.5 T	0, 50, 500	Manual ROI	Within 3 months before and after TACE	Adriamycin microspheres (embospheres)

Ou et al. [30]	Total, 21; HBV, 11/21; HCV, 4/21; HCV and HBV, 4/21; alcohol, 1/21; and other, 1/21	21	16	5	GE healthcare, 1.5 T	0, 400	Manual ROI	1, 2, 4 weeks	DEB vials up to 50 mg doxorubicin ×2

Yuan et al. [31]	Total, 43 and acute or chronic viral hepatitis, 32/43	59	36	23	Philips 3 T	0, 800, 1,500, 2,000	Manual ROI	1 month, every 3 months	1–1.5 g 5FU, 30–40 mg hydroxycamptothecin, 40–50 mg Adriamycin, 3–20 mL Lipiodol, gelatin sponge particles

HBV, hepatitis B virus; HCV, hepatitis C virus; ADC, apparent diffusion coefficient; TACE, transcatheter arterial chemoembolization; ROI, region of interest; DEB, drug-eluting bead.

**Table 2:** Relative ΔADC of responder and nonresponder lesions post-TACE

	Responder		Nonresponder	
	*n* (%)	relative change, %	*n* (%)	relative change, %
Kokabi et al. [26]	6 (3.3)	35.6	6 (5.4)	6.6
Kubota et al. [27]	23 (12.6)	85.2	13 (11.7)	8.0
Lin et al. [28]	67 (36.8)	30.7	51 (45.9)	73.2
Mannelli et al. [29]	34 (18.7)	17.1	13 (11.7)	−0.7
Ou et al. [30]	16 (8.8)	23.6	5 (4.5)	0.7
Yuan et al. [31]	36 (19.8)	24.8	23 (20.7)	4.1
Summary	182 (100)	33.4	111 (100)	35.7

ΔADC, apparent diffusion coefficient value change; TACE, transcatheter arterial chemoembolization.

## Notes

Ralph Drewes, Constanze Heinze, Alexey Surov, and Jazan Omari contributed equally to this work.
